# A novel thiazolidinediones ATZD2 rescues memory deficits in a rat model of type 2 diabetes through antioxidant and antiinflammation

**DOI:** 10.18632/oncotarget.22467

**Published:** 2017-11-18

**Authors:** Xuan-Kang Wang, Ting Sun, Yu-Jiao Li, Yu-Hong Wang, Yan-Jiao Li, Liu-Di Yang, Dan Feng, Ming-Gao Zhao, Yu-Mei Wu

**Affiliations:** ^1^ Department of Pharmacology, School of Pharmacy, The Fourth Military Medical University, Xi’an, Shaanxi Province 710032, P.R. China; ^2^ Student Brigade, The Fourth Military Medical University, Xi’an, Shaanxi Province 710032, P.R. China; ^3^ Department of Emergency, PLA Army General Hospital, Beijing 100700, P.R. China; ^4^ Department of Acupuncture-Moxibustion-Massage, Shaanxi University of Chinese Medicine, Xi’an, Shaanxi Province 712000, P.R. China; ^5^ Department of Diagnostic Radiology, Tangdu Hospital, The Fourth Military Medical University, Xi’an, Shaanxi Province 710032, P.R. China

**Keywords:** diabetes, peroxisome proliferator-activated receptor γ (PPAR-γ), thiazolidinediones, rosiglitazone, neuroprotection

## Abstract

Type 2 diabetes (T2DM) has been associated with learning and memory impairment; however, drugs for diabetes could not prevent the development of cognitive decline in T2DM patients. In the present study, compounds derived from thiazolidinediones (TZD), a PPAR-γ agonist, were synthesized by conjuncting the alkyl-substituted benzimidazole group to TZD group (ATZDs). Based on the *in vitro* evaluation, the neuroprotection of ATZD2 was further investigated using a streptozotocin-induced T2DM rat model. Pharmacokinetic study showed that ATZD2 could pass the blood-brain barrier (BBB) while the rosiglitazone (RSG, the precursor compound of ATZD2) not. Administration of ATZD2 significantly promoted the survival rate and attenuated fasting blood glucose (FBG) levels as compared to RSG treatment in T2DM rats. Furthermore, ATZD2 treatment ameliorated the impairment of learning and memory by Morris water maze test. The beneficial effects of ATZD2 were associated with the down-regulation of hypoxia induced factor-1α, aldose reductase, and Bax expression which are related to T2DM pathology. ATZD2 treatment also attenuated the expression of inflammatory cytokines and restored the balance of redox in the diabetic hippocampus. These effects were more potent as compared with that of RSG at the same dose. The data indicate that ATZD2 may be a potent agent for the treatment of cognitive dysfunction in T2DM.

## INTRODUCTION

Type 2 diabetes mellitus (T2DM), resulting from defective insulin secretion, resistance to insulin action or both [1], is associated with long-term complications affecting the eyes, kidneys, heart and nerves. Diabetes causes a variety of structural and functional disorders in the central and peripheral nervous systems including neuropathy, cerebral atrophy, cognitive dysfunction, and an increased risk of dementia [2–4]. Diabetes-related cognitive dysfunction is largely a consequence of changes within the central nervous system (CNS) that are secondary to chronic hyperglycemia. A series of neuropathological and neurobehavioral changes in diabetic patients are called as central diabetic encephalopathy or neuropathy [5]. Abnormalities in electrophysiological and structural of brains may be responsible for impaired cognitive functions in diabetes mellitus [1].

The mechanisms underlying diabetic encephalopathy appear to be a multi-factorial process. Evidence shows that the cerebrovascular changes, oxidative stress [6, 7], increased advanced glycation end products (AGE) [8], inflammatory cytokines [9], and impairments in cerebral insulin signaling systems are underlying the diabetic encephalopathy. Moreover, aldose reductase (AR), a NADPH-dependent enzyme belonging to aldehyde-keto reductase superfamily, plays a critical role in diabetes mellitus by providing potent precursors of AGE [10] and altering the cellular redox balance. The increase in AGE formation and the aldose reductase-polyol pathway flux are the main mechanisms recruited by oxidative stress [11]. However, at present, no specific treatments are available for the management and/or prevention of cognitive dysfunction in T2DM.

Thiazolidinediones (TZDs) are synthetic agonists for peroxisome proliferator-activated gamma-type (PPAR-γ) receptor which are used clinically for the treatment of T2DM. TZDs have been shown to reestablish insulin sensitivity, improve lipid profiles, and reduce inflammation and oxidative stress [12, 13]. Furthermore, TZDs exert multiple beneficial effects on age-related cognitive decline [14, 15], reestablishing glucose regulation and normal lipid levels, reducing inflammation and amyloid-β (Aβ) peptide, as well as increasing cerebral blood flow in humans [14, 15]. Rosiglitazone (RSG), one of the widely-used TZDs, is beneficial for part of diabetic patients. However, clinical data show that RSG increases the risks of myocardial infarction and death from cardiovascular causes [16]. The improvement in learning and memory of RSG in T2DM is also limited due to the poor penetration into the brain [17]. It is urgent to explore new agents to meet the challenges in diabetic encephalopathy therapy.

In order to improve the pharmacological activities of RSG in CNS, we synthesized a serial of TZD derives named as ATZD2, ATZD4 and ATZD6, in which alkyl-substituted benzimidazole group has low hydrophilic, high liposolubility and biocompatibility [18, 19]. Further analysis showed that ATZD2 promoted the survival rate of T2DM rats, ameliorated diabetes-induced impairment of learning and memory, and prevented the development of cataract in T2DM rats. The beneficial effects of ATZDs may include penetration into the brain, reestablishing glucose homeostasis, and the rebalancing oxidative stress and redox signaling in T2DM rats.

## RESULTS

### Synthesis of benzimidazole derivative compounds

**Figure 1:**
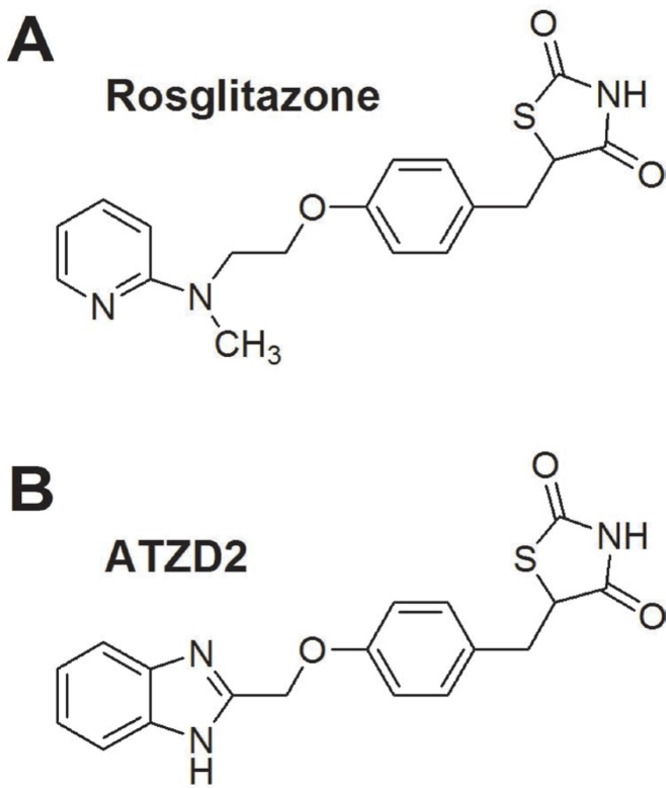
The structures of compounds (**A**) Structure of Rosiglitazone (RSG). (**B**) Structure of ATZD2.

Serial compounds derived from thiazolidinediones (TZD) were synthesized by conjuncting the alkyl-substituted benzimidazole group to TZD group. The chemical structure of ATZD2 was confirmed by hydrogen nuclear magnetic resonance (^1^HNMR) [18, 19]. ATZD2 was selected as the target drug from the serial synthesized compounds based on the better neuroprotective effects *in vitro* (data not shown). The structures of precursor rosiglitazone (RSG) and compound ATZD2 were showed in Figure 1.

### Pharmacokinetic properties of ATZD2

**Figure 2:**
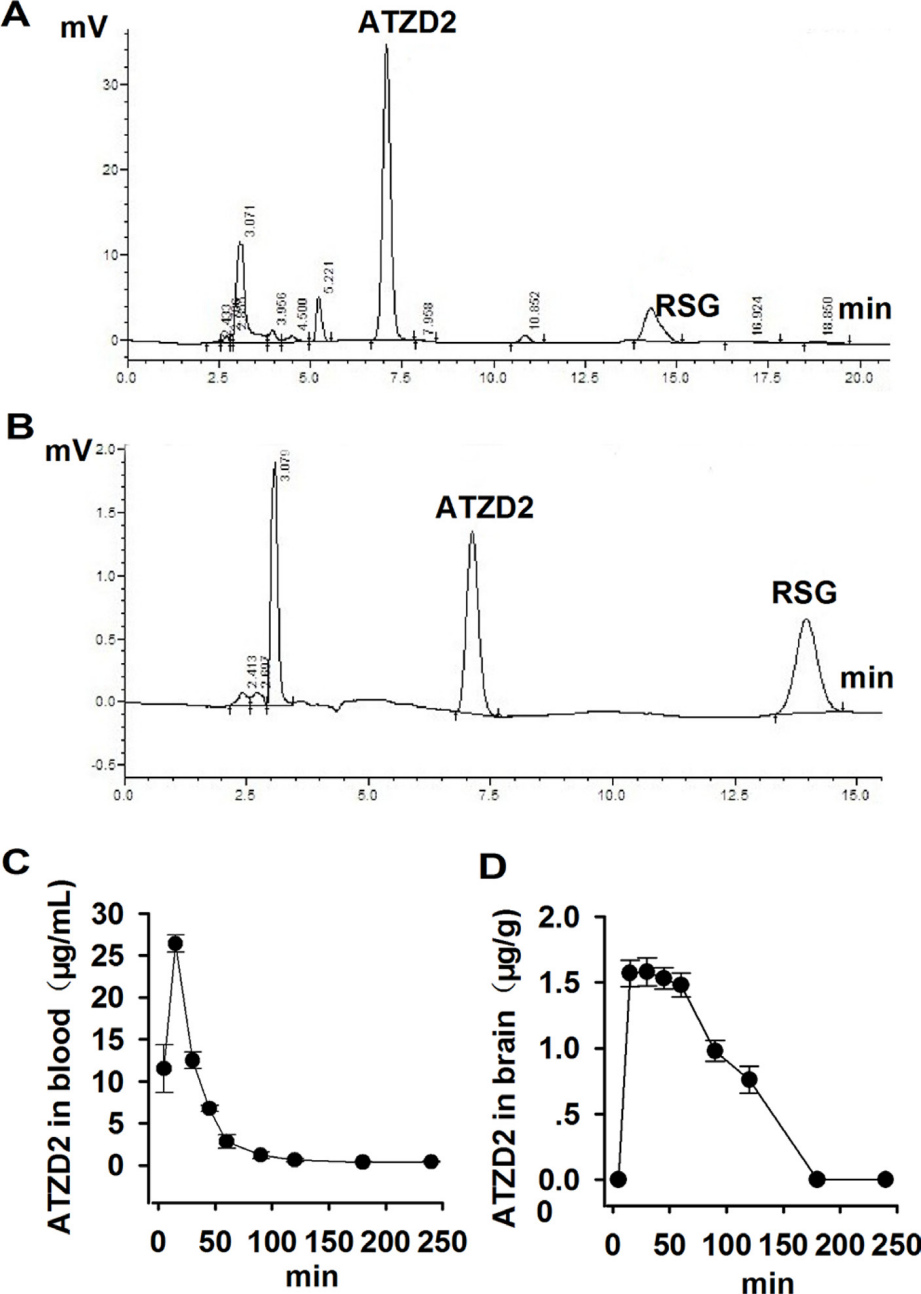
The pharmacokinetic study of compound ATZD2 Compound ATZD2 was administrated to normal rats by *i.p* injection at 25 mg/kg, precursor rosiglitazone (RSG) was used as the internal standard. HPLC-UV method was used. Compound ATZD2 was separated well from internal standard RSG in blood (**A**) as well as in brain (**B**) using this working system. Orbital blood was collected at 0, 5 min, 0.25, 0.5, 1.0, 1.5, 2.0, 3.0 and 4.0 h after ATZD2 administration, and the concentrations of ATZD2 in blood (**C**) and brain (**D**) were determined.

High Performance Liquid Chromatography-UV (HPLC-UV) method was used to determine the pharmacokinetic process of ATZD2 in rats. ATZD2 (25 mg/kg) was administrated by injection intraperitoneally (*i.p*). Orbital blood was collected at 0, 5 min, 0.25, 0.5, 1.0, 1.5, 2.0, 3.0 and 4.0 h after ATZD2 administration. The concentrations of ATZD2 in blood and brain were detected. Precusor RSG was used as the internal standard. Compound ATZD2 was separated well from internal standard RSG in blood (Figure 2A) as well as in brain (Figure 2B) using the same working system as described in the methods.

The concentration of ATZD2 in plasma was 26.4 μg/mL after administration for 5 min (Figure 2C), and 1.57 μg/g in the brain at the same time point (Figure 2D), implicating that ATZD2 could pass through the blood-brain barrier (BBB). It has been reported that the concentration of RSG, the precursor of ATZD2, in the cerebrospinal fluid was only 0.0045% of that in blood [17]. In present study, we failed to detect RSG in brain tissue other than in blood using the same method as that of ATZD2, maybe due to the different method used. Present data indicate that ATZD2 can pass through the BBB into the brain more easily than RSG, showing the more beneficial effects than RSG to target on the nervous system.

### Effects of ATZD2 on blood glucose levels in T2DM rats

**Figure 3:**
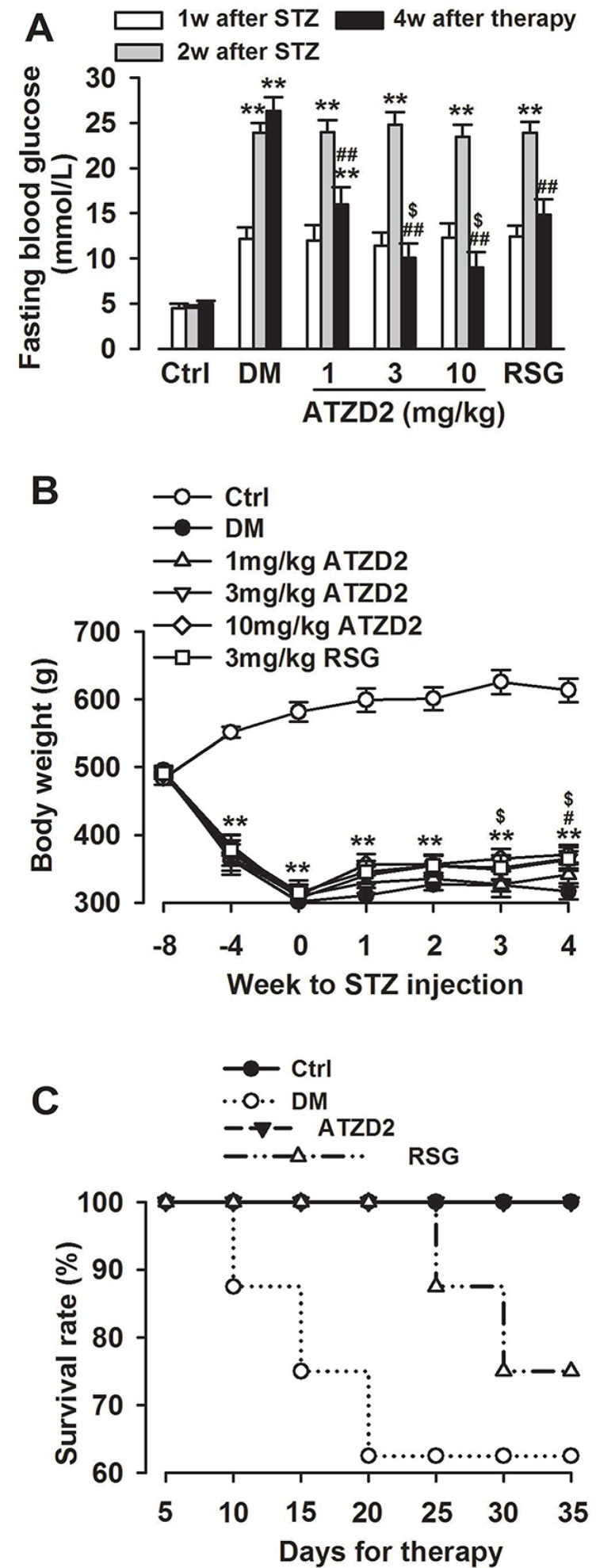
General effects of ATZD2 on T2DM rats (**A**) Effects of ATZD2 (1, 3, 10 mg/kg) and rosiglitazone (RSG, 3 mg/kg) on the fasting blood glucose level. (**B**) Effects of ATZD2 and RSG on the body weight. (**C**) Effects of ATZD2 (3 mg/kg) and RSG (3 mg/kg) on survival rate. *n* = 12 in each group, ^**^P < 0.01 compared with the normal control group; *^##^P* < 0.01 compared with the T2DM group; *^$^P* < 0.05 compared with the RSG-treated group.

Streptozotocin (STZ)-induced diabetes is a well-documented model for experimental diabetes. In present study, STZ injection successfully induced diabetes mellitus with defects in insulin sensitivity and secretion. The fasting blood glucose (FBG) reached 12.18 ± 2.25 mmol/L one week after STZ injection, which was significantly higher compared to non-diabetic controls (4.45 ± 0.15 mmol/L; *P* < 0.001; Figure 3A). The increased glucose level and the diabetic symptoms persisted during the experimental period. Chronic treatment with ATZD2 (1, 3, 10 mg/kg) for 4 weeks significantly reduced FBG levels in diabetic rats to 15.94 ± 1.96, 15.04 ± 1.60 and 13.99 ± 1.68 mmol/L respectively, when compared with vehicle treatment in diabetic rats (26.32 ± 3.49 mmol/L, *P* < 0.01). The FBG level was also found decreased to 14.83 ± 1.74 mmol/L in RSG-treated group (3 mg/kg), compared with the T2DM group (*P* < 0.01, Figure 3A).

T2DM rats also showed a continued decline in body weight (Figure 3B), accompanied with polydipsia, polyuria and polyphagia which are observed in diabetes patients. The body weight of STZ-diabetic rats was significantly lower at 21 d after injection of STZ than that of controls (control group, 625.9 ± 17.8 g; T2DM, 325.4 ± 10.7 g). ATZD2 treatment prevented loss of body weight compared with T2DM rats (ATZD2 group, 370.8 ± 14.2 g; T2DM group, 316.5 ± 12 g) after 4 weeks treatment (Figure 3B). Last, the survival rate of T2DM was evaluated. ATZD2 treatment promoted the diabetic rat survival rate similar to the normal control, whereas the survival rate of diabetic-rats declined to 62.5% at the end of study. RSG administration promoted the diabetic-rats survival rate to 75% (Figure 3C).

### ATZD2 treatment rescues the deficits of learning and memory in T2DM rats

**Figure 4:**
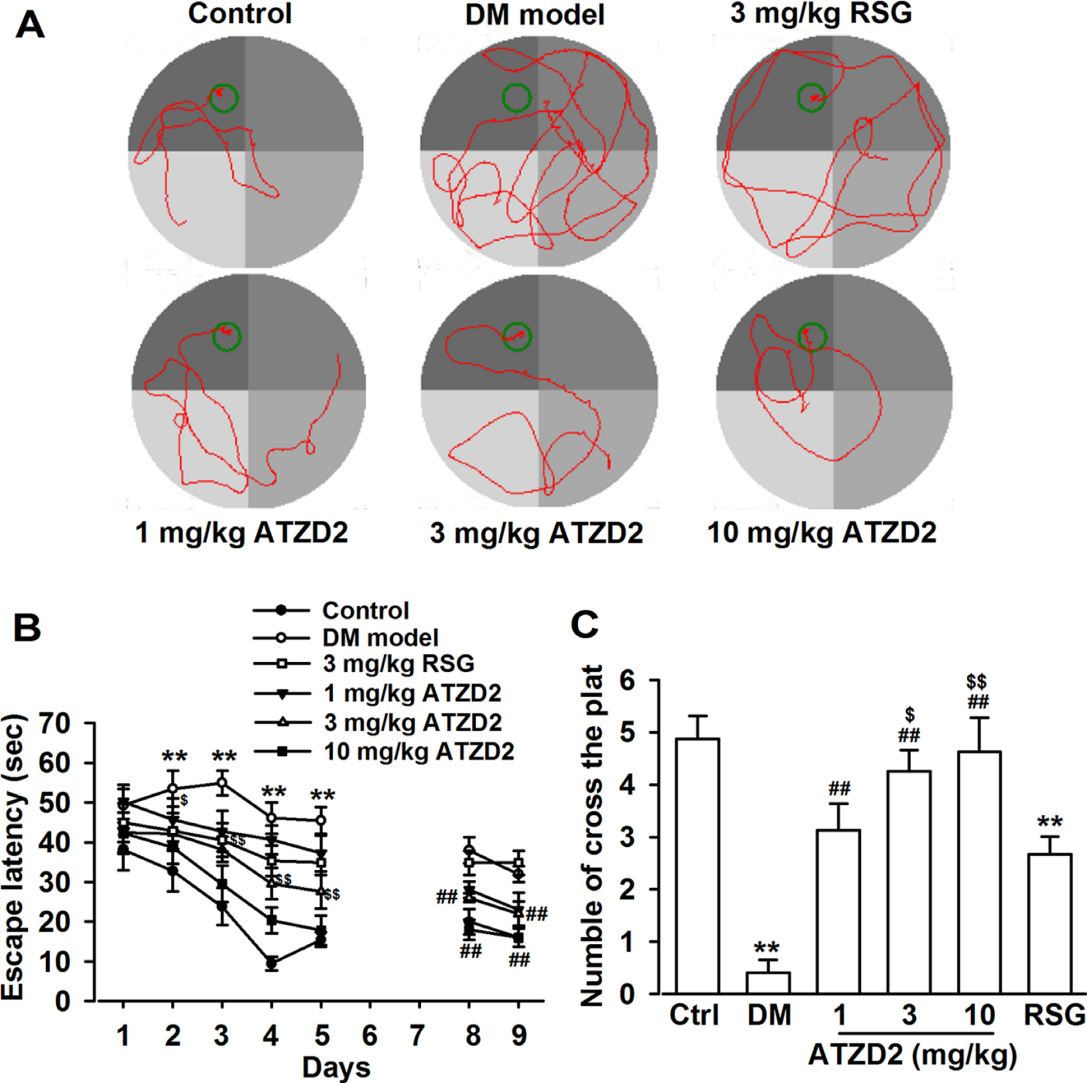
ATZD2 attenuates learning and memory deficits in T2DM rats (**A**) Sample traces of directional navigation in the Morris water maze test from each group. (**B**) Effects of ATZD2 (1, 3, 10 mg/kg) and rosiglitazone (RSG, 3 mg/kg) on the mean escape latency over 5 consecutive days of training and testing on day 8–9. (**C**) Effects of ATZD2 and RSG on the number of cross the platform. *n* = 12 in each group, ^**^P < 0.01 compared with the normal control group; *^##^P* < 0.01 compared with the T2DM group; *^$^P* < 0.05, *^$$^P* < 0.01 compared with the RSG-treated group.

The cognitive function was assessed using Morris water maze (MWM) test. The mean escape latency for trained rats was decreased during the 20 learning trials in all the groups (Figure 4A). Control diabetic rats (vehicle treated) exhibited significantly higher escape latency on day 2–5 during training trials compared with vehicle-treated nondiabetic rats; whereas ATZD2 (1, 3, and 10 mg/kg) dose-dependently decreased the escape latencies in diabetic rats on day 2–5 (Figure 4A, 4B). Chronic treatment with RSG (3 mg/kg) in diabetic rats showed comparable effects to the same dose of ATZD2. The performance of all the groups in the trial with the visible platform was not significantly different (data not shown). Treatment of ATZD2 (1, 3, and 10 mg/kg) significantly prevented the memory impairment as indicated by the increased time spent in target quadrant. Furthermore, the effect of ATZD2 on the platform-crossing experiment at 3 mg/kg was much better than the same dose of RSG treatment (Figure 4C). No significant difference was observed in the swimming speed throughout the test period in each group.

### Effects of ATZD2 on the expression levels of aldose reductase and hypoxia inducible factor-1α in T2DM rats

**Figure 5:**
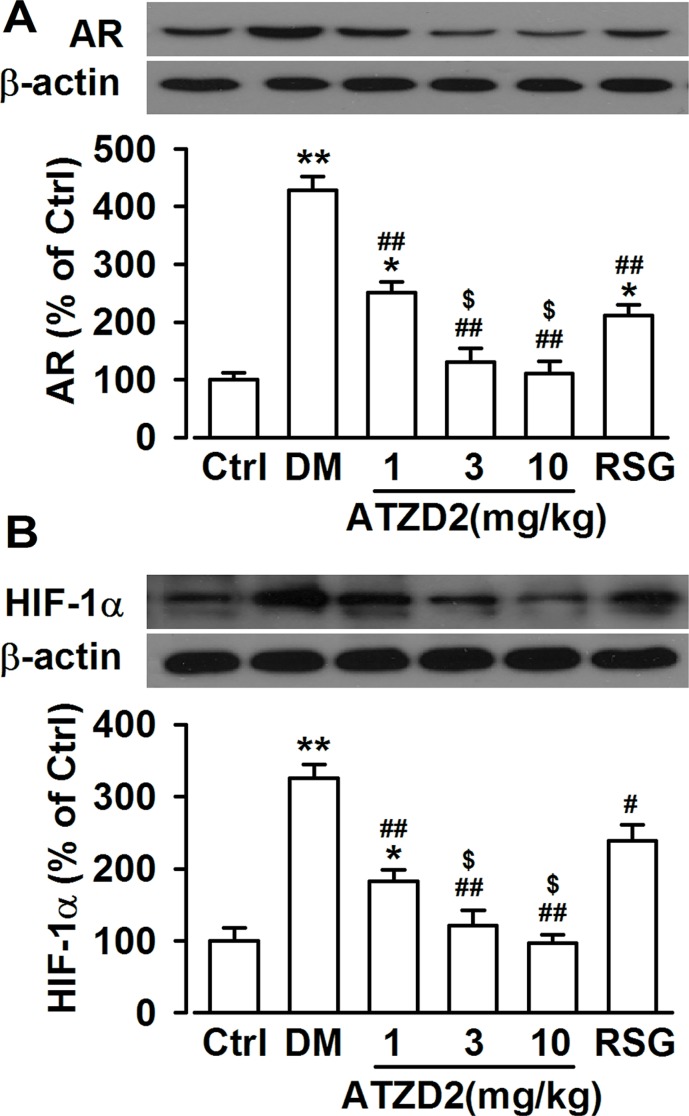
Effects of ATZD2 on aldose reductase (AR) and hypoxia induced factor 1α (HIF-1α) in T2DM rats (**A**) Upper: Representative Western blot results showing levels of aldose reductase (AR) protein; Lower: effects of ATZD2 (1, 3, 10 mg/kg) and rosiglitazone (RSG, 3 mg/kg) on the intensity of the AR normalized to β-actin in the brain. (**B**) Upper: Representative Western blot results showing levels of hypoxia induced factor 1α (HIF-1α) protein; Lower: effects of ATZD2 and RSG on the intensity of the HIF-1α normalized to β-actin in the brain. *n* = 12 in each group, ^*^P < 0.05, ^**^P < 0.01 compared with the normal control group; *^#^P* < 0.05, *^##^P* < 0.01 compared with the T2DM group; *^$^P* < 0.05 compared with the RSG-treated group.

Aldose reductase (AR), the first rate-limiting enzyme in the polyol pathway, is well known to contribute to oxidative stress in the DM [20]. As a NADPH-dependent enzyme, the subsequent high consumption of cellular NADPH impedes the regeneration of glutathione and alters the cellular redox balance, thus increasing the susceptibility to oxidative stress. Therefore, inhibition of AR in diabetes may protect against damage in the brain. Levels of AR significantly increased in hippocampus of T2DM rat; however, ATZD2 dose-dependently decreased the expression levels of AR (Figure 5A). This effect of ATZD2 (3 mg/kg) was much more effective than that of the same dose of RSG. Transcription factor hypoxia inducible factor-1α (HIF-1α)-mediated signaling has been implicated in both cell survival and cell death pathways [21]. Since hypoxia may mediate diabetic encephalopathy progression, we further detected HIF-1α level in the hippocampus. ATZD2 administration dose-dependently reversed the enhanced level of HIF-1α in T2DM rats and this effect was more potent than that of RSG at the same dose (Figure 5B).

### Effects of ATZD2 on lipid peroxidation in diabetic rats

**Figure 6:**
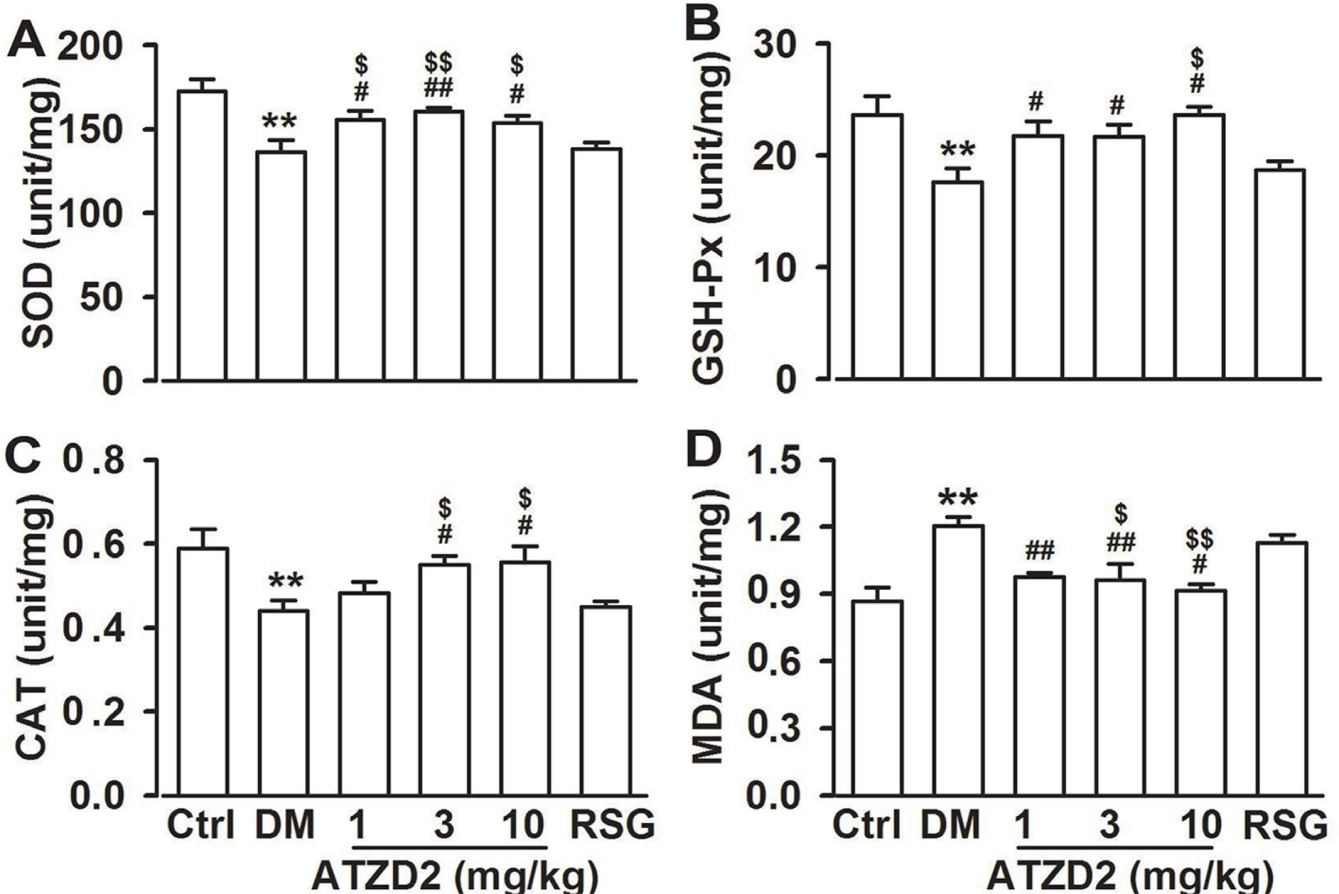
Effects of ATZD2 on lipid peroxidation in rat brains Effects of ATZD2 (1, 3, 10 mg/kg) and rosiglitazone (RSG, 3 mg/kg) on the activities of superoxide dismutase (SOD, **A**), glutathione peroxidase (GSH-Px, **B**), catalase (CAT, **C**) and levels of malondialdehyde (MDA, **D**) in the brain homogenates. *n* = 8 in each group, ^**^P < 0.01 compared with the normal control group; *^#^P* < 0.05, *^##^P* < 0.01 compared with the T2DM group; *^$^P* < 0.05, *^$$^P* < 0.01 compared with the RSG-treated group.

Imbalance between the production of reactive oxygen species/reactive nitrogen species (ROS/RNS) as a consequence of mitochondrial dysfunction and the intracellular antioxidant capacity leads to abnormally elevated ROS levels called as oxidative stress that is followed by oxidative damage to cells and, eventually, death [22]. Next, effects of ATZD2 on the balance of redox including superoxide dismutase (SOD), glutathione peroxidase (GSH-Px), catalase (CAT) and levels of malondialdehyde (MDA) in diabetic brains were detected. Compared with normal group, the activities of SOD, CAT and GSH-Px decreased, and MDA levels increased significantly in brain homogenates of T2DM rats (Figure 6A). Chronic administration of ATZD2 significantly increased the levels of SOD (Figure 6A), GSH-Px (Figure 6B), and CAT (Figure 6C), and decreased the levels of MDA (Figure 6D). However, administration of RSG had no effects on the alteration of SOD, GSH-Px, CAT, and MDA in the brains of T2DM rats. The data indicated the antioxidant activities in the brain of T2DM animals.

### Effects of ATZD2 on the levels of inflammatory cytokines

**Figure 7:**
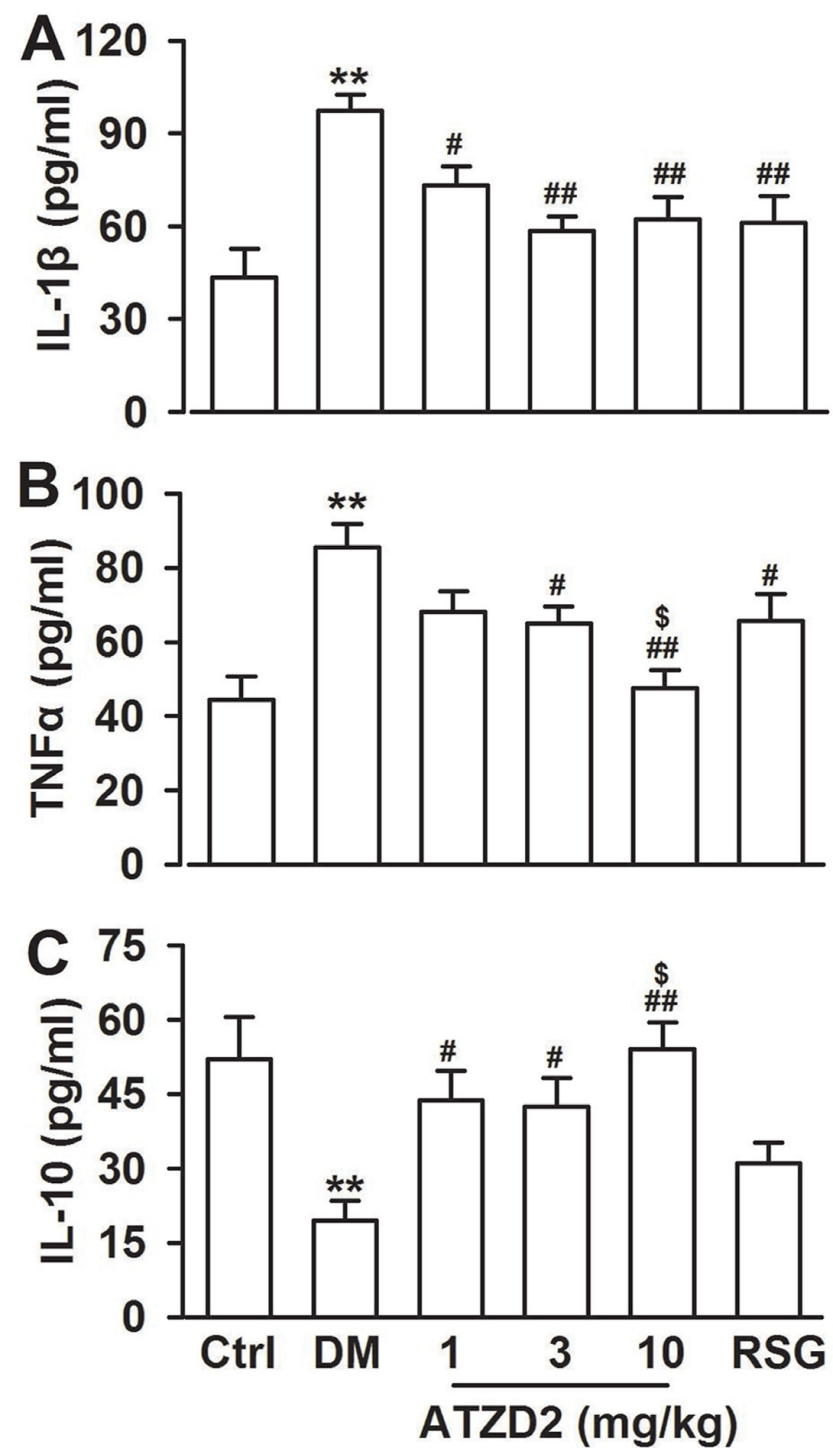
Effects of ATZD2 on the inflammatory cytokines Effects of ATZD2 (1, 3, 10 mg/kg) and rosiglitazone (RSG, 3 mg/kg) on the expression levels of IL-1β **A**, TNF-α **B**, and IL-10 **C** in the blood of diabetic rats. *n* = 8 in each group, ^**^P < 0.01 compared with the normal control group; *^#^P* < 0.05, *^##^P* < 0.01 compared with the T2DM group; *^$^P* < 0.05 compared with the RSG-treated group.

Proinflammatory cytokines TNF-α, IL-1β, and IL-6 are increased in T2DM and the accumulation of cytokines induce neuronal death by increasing apoptosis, reducing synaptic activity, and inhibiting neurogenesis, thus contribute to the impaired cognition [23]. Last, levels of inflammatory cytokines IL-1β and TNF-α and anti-inflammatory cytokine IL-10 were detected in the plasma, since inflammation is a key pathogenic factor for diabetes consequence [24]. The levels of IL-1β and TNF-α in T2DM group were increased significantly, while levels of IL-10 were decreased significantly in T2DM rats (Figure 7). Chronic administration of ATZD2 significantly decreased the levels of IL-1β (Figure 7A) and TNF-α (Figure 7B) and increased the levels of IL-10 (Figure 7C) in a dose-dependent manner. Same dose of RSG (3 mg/kg) induced a comparable effect with ATZD2.

### ATZD2 inhibits apoptosis in the hippocampus of T2DM

**Figure 8:**
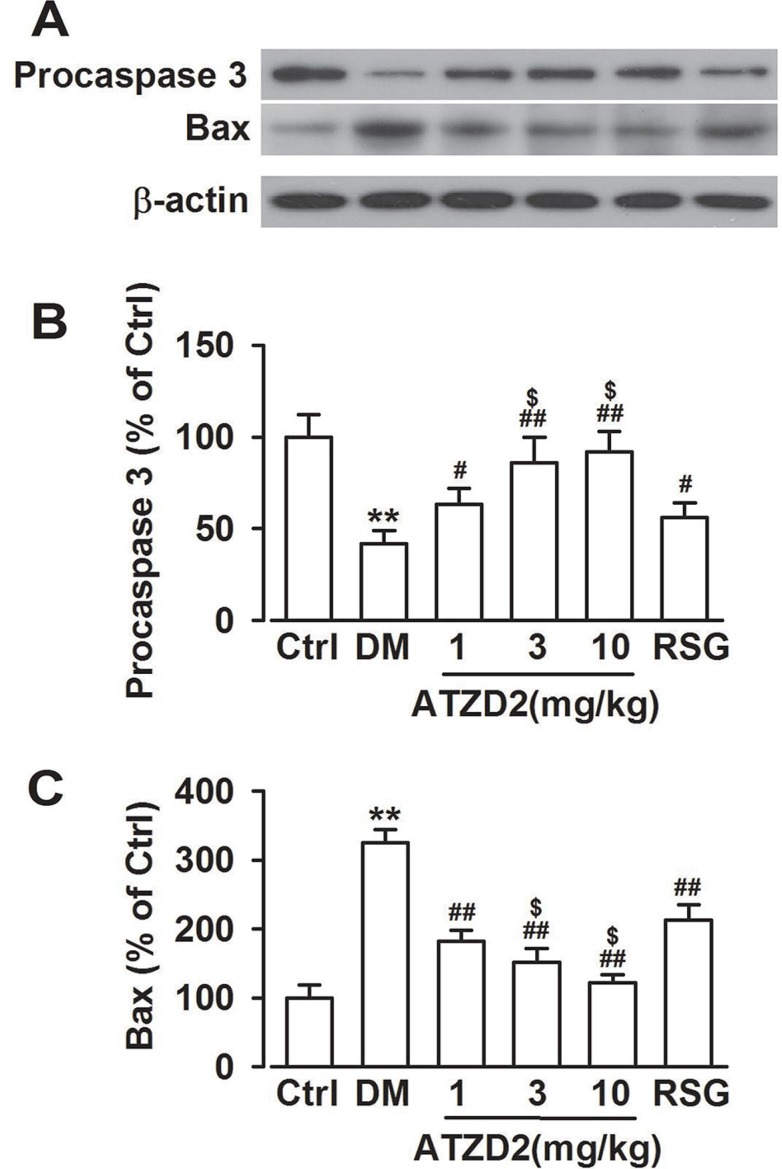
Effects of ATZD2 on the apoptosis in the hippocampus of diabetic rats (**A**) Representative Western blot results showing levels of Procaspase-3 and Bax. (**B**) Effects of ATZD2 (1, 3, 10 mg/kg) and rosiglitazone (RSG, 3 mg/kg) on the intensity of the Procaspase-3 normalized to β-actin in the brain. (**C**) Effects of ATZD2 and RSG on the intensity of the Bax normalized to β-actin in the brain. *n* = 12 in each group, ^**^P < 0.01 compared with the normal control group; *^#^P* < 0.05, *^##^P* < 0.01 compared with the T2DM group; *^$^P* < 0.05 compared with the RSG-treated group.

Apoptosis regulatory proteins have been implicated in the susceptibility of neurons to cell death in the diabetic brain. Western blot analysis revealed that the levels of procaspase-3 (the precursor of caspase-3) and Bax in the hippocampus were significantly altered in the T2DM model rats. Levels of Bax were significantly increased, whereas procaspase-3 protein levels were significantly decreased (Figure 8). ATZD2 administration reversed the alteration of Bax and procaspase-3, which may contribute to the cell viabilities. These data indicate the anti-apoptotic activities of ATZD2 in T2DM models.

## DISCUSSION

In the present study, neuroprotective effects of ATZD2, the compound by conjuncting the alkyl-substituted benzimidazole group to TZD group, were evaluated in the T2DM model animals. As compared with the precursor compound RSG, ATZD2 could pass through the BBB and showed more beneficial activities against the injuries in the T2DM animals. Chronic treatment of ATZD2 promoted survival rate and improved learning and memory in T2DM rats. The underlying mechanisms involved in the inhibition of apoptosis, lipid peroxidation, and inflammation response.

Recently, the brain is recognized as a target of end-organ damage in diabetes [27]. Numerous literatures suggest that diabetes-related cognitive dysfunction is largely a consequence of changes secondary to chronic hyperglycemia within the CNS [28, 29]. Rosiglitazone, a PPAR-γ agonist, is shown to be neuroprotective in focal cerebral ischemia and intracerebral hemorrhage. RSG is a constituent of the thiazolidinediones nuclear hormone receptor superfamily and is known for its anti-inflammatory actions via activation of PPAR-γ. The activation of PPAR-γ leads to the inhibition of the inflammatory NF-kB pathway [12, 13]. Thus, RSG attenuates the expression of pro-inflammatory genes and cytokine production by regulating ligand activation of transcription factors. RSG's neuroprotection is limited due to the poor penetration into the brain [17], even though report has shown the ability of RSG to penetrate the blood-brain barrier.

A well-documented model of experimental diabetes in rat was induced by streptozotocin (STZ) injection. Antihyperglycemics and insulin sensitizers reduced cognitive dysfunctions in diabetic condition [25]. Chronic treatment with synthesized ATZD2 restored the body weight and FBG level in T2DM rats. These activities are beneficial for promoting survival rate and ameliorating cognitive deficits of T2DM rats. As a derivate from TZD, ATZD2 is more effective than RSG in the promoting survival rate and ameliorating cognitive deficits, even though the activity of reducing blood glucose levels is comparable between ATZD2 and RSG. Thus, the detailed mechanisms underlying potent neuroprotection of ATZD2 are needed to explore.

Aldose reductase is the rate-limiting enzyme of the polyol pathway. Enhanced AR activity leads to the reduction of glutathione synthesis, impairment of nitric oxide synthesis, reduction of Na^+^, K^+^-ATPase activity, increase of protein kinase C activity, as well as the redox imbalance. All of these alterations have been identified participating in the development of diabetic complications including central diabetic encephalopathy [30–32]. Transcription factor HIF-1α-mediated signaling has been implicated in both cell survival and cell death pathways [21]. Increased mRNA and protein expression of HIF-1α are found in diabetic rats [33]. Elevated serum HIF-1α may be involved in vascular calcification in patients with type 2 diabetes mellitus [34]. Levels of AR and HIF-1α were significantly decreased in ATZD2-treated diabetic rats, suggesting the therapeutic activity of ATZD2 correlated with regulating the HIF-1α and AR pathways. The more potent effects of ATZD2 than RSG may partially explain the more effective of ATZD2 in the promoting survival rate and ameliorating cognitive deficits.

Reactive oxygen species/reactive nitrogen species (ROS/RNS) play a dual biological role in living systems based on different levels. Increased production of ROS/RNS or decreased antioxidant capacity as a result of increased carbohydrate metabolism in insulin target tissues may alter the phosphorylation status of these signalling nodes causing deactivation, and the accumulation of oxidatively damaged proteins, lipids, and nucleic acids correlates with the onset of age-related cellular alterations, especially in diabetes [7, 35–37]. The high content of lipids, high requirement for oxygen, and the scarcity of antioxidant defence mechanisms make the brain highly susceptible to oxidative stress. An increase in reactive species production due to oxidative stress seems to play a key role in neuronal damage. Oxidative damage to the synapse in the cerebral cortex and hippocampus has been previously reported contributing to the deficit of cognitive functions [38]. In addition, it is reported that antioxidants prevent the diabetes-induced cognitive dysfunction [29]. In the present study, lipid peroxidation levels were significantly increased, whereas reduced glutathione activity was markedly decreased in diabetic rats, which is well in accordance to earlier reports. Treatment with ATZD2 returned the levels of lipid peroxides, reduced glutathione and restored the activities of GSH-Px, CAT, SOD and MDA level towards their control values in diabetic brains.

Evidence showed that increased inflammatory cytokines, which were derived in adipose tissue, linked with insulin resistance. Insulin signalling, rich in hippocampus, is increasingly recognised to modulating memory and learning, and being required for synaptic plasticity, as well as neuroprotective properties [39, 40]. Thus, insulin resistance contributes to cognitive dysfunction in diabetes [41]. Furthermore, inflammation is a crucial process underlying memory and cognitive function [42]. Excess TNF-α is toxic to cortical neurons. It is shown that the over-expression of TNF-α in transgenic mice consistently results in severe inflammation, encephalopathy, and neurodegeneration [42]. In present study, inflammatory cytokines increased markedly in the plasma of diabetic rats. ATZD2 might ameliorate inflammation responses in T2DM rats.

In conclusion, the neuroprotective effects of ATZD2 observed in the present study could be multivariate. The findings suggest that beneficial effects of ATZD2 may be attributed to its anti-oxidant, inhibition of AR activity, anti-apoptosis, and restoration the redox balance. These effects of ATZD2 were more potent as compared to its precusor RSG at same dose, suggesting that ATZD2 may be a potent agent for the treatment of cognitive dysfunction in T2DM.

## MATERIALS AND METHODS

### Reagents and antibodies

Streptozotocin (STZ), anti-aldose reductase (AR) and anti-β-actin antibodies were purchased from Sigma-Aldrich (St. Louis, MO, USA). Anti-hypoxia-induced factor 1α (HIF-1α), Anti-microtubule-associated proteins 2 (MAP2), Anti-Bcl-2-associated X protein (Bax), and Anti-Procaspase-3 antibodies were purchased from Chemicon (Temecula, CA, USA). All secondary antibodies conjugated with horseradish peroxidase (HRP) were purchased from Santa Cruz Biotechnology (Santa Cruz, CA, USA). Kits for malondialdehyde (MDA), superoxide dismutase (SOD), catalase (CAT), and glutathione peroxidase (GSH-Px) were purchased from Nanjing Jiancheng Bioengineering Institute (Nanjing, China). ELISA Kits for TNF-α, IL-1β, and IL-10 were purchased from R and D Systems (Minneapolis, MN, USA). Other chemicals and reagents were from Sigma unless mentioned. All of the chemicals and reagents used were commercially available and of standard biochemical quality. All the drugs were dissolved in double distilled water except streptozotocin (STZ), which was dissolved in citrate buffer (pH 4.4).

### Animals

Adult male Sprague-Dawley (SD) rats (body weight 200–220 g) were purchased from the Experimental Animals Center of the Fourth Military Medical University (certificate No. 201000082, Grade II). Rats were housed in colony cages (three rats per cage) and maintained at 23 ± 2°C under 12 h-light and 12 h-dark cycle with food and water *ad libitum*. The animals were allowed to acclimatize to the laboratory environment for 1 week and then randomly divided into various groups. Animal care and procedures were approved by the Institutional Animal Care and Use Committee of The Forth Military Medical University in full compliance with international rules and policies. Efforts were made to minimize the number of animals used and their suffering. All tests were conducted between 9:00 am and 1:00 pm.

### Induction of rat diabetes

The rats were randomly divided into challenge and normal control groups after acclimatization for 1 week. Defect in insulin sensitivity and secretion model, namely high-fat diet (HFD) STZ-induced diabetic rats, was used in this study. HFD consisted of 10% fat, 20% sucrose, 2.5% cholesterol, 1% cholate, 1% egg, 30% bean sprout, and 35.5% normal diet. Rats in normal control group were fed with normal laboratory diet as before. After 4 weeks of feeding HFD, the animals were fasted for 12 h and were injected *i.p* with a single dose of STZ (30 mg/kg in 0.1 M sodium citrate buffer, pH 4.4), while the control rats were given an equivalent volume of vehicle. STZ-treated rats received 5% of glucose solution instead of water for 24 h after injection to reduce death due to hypoglycemic shock. The FBG level from tail vein was measured after STZ administration for 3 d, and 60 rats with FBG level above 7.8 mmol/L were considered as diabetic, 12 rats for each group and there were 5 groups used for the further studies. HFD was continued throughout the study.

### Treatments schedule

Diabetic model rats were administered orally with ATZD2 (1, 3, or 10 mg/kg), RSG (3 mg/kg), or vehicle (1 ml/kg), once daily for the next 30 d (day 1–30). At the end of treatment, rats were subjected to Morris water maze test (day 31–36). Similar treatments were given to control (non-diabetic) rats.

### Morris water maze test

Morris water maze test was performed as previously described [25]. MWM is a circular and galvanized water tank (180 cm diameter × 60 cm height) made up of dark gray plastic that is partially filled with water. The water was made opaque by the addition of milk powder, and the temperature was adjusted to 24 ± 1°C. The surface area of the tank was divided into four equal quadrants. An escape platform (12.5 cm in diameter and 38 cm in high) was placed in one of the four maze quadrants (the target quadrant) submerged 2.0 cm below the water surface (30 cm away from the side wall). The platform remained in the same quadrant during the entire experiment. Rats were required to find the hidden platform using only distal spatial cues available in the testing room. The cues were maintained constant throughout the experiments. The rats were released gently into the water, always facing the tank wall. A different starting position was used on each trial. They were given 90 s to find the platform. Rats were allowed to remain on it for 20 s when reaching the platform. They were taken out, dried, and placed in a separate cage for 1 h before the next trial. The rats received four training trials daily for 5 consecutive d (day 31–35 after diabetes induction) to locate and escape onto the platform, and the spatial learning scores (latency in seconds) were recorded. Between the trials, the water was stirred to erase olfactory traces of previous swim patterns. The escape platform was kept in the same position relative to the distal cues. If the rat failed to locate the platform within allowed time of 90 s, it was assisted by the experimenter and allowed to stay there for the same period of time. The time to reach the platform (latency in seconds) was measured. Swimming was video-tracked. The latency, path length, swim speed, and cumulative distance to the platform were recorded. Mean swim latency for all trials on each day in each group was determined and compared between groups.

To test possible deficits in sensorimotor processes, rats were tested in the water maze with a visible platform on a new location on the final day of training. The test with the visual platform does not require special orientation [26] and was used to show possible deficits in sensorimotor processes. For the test, black target platform was placed inside the pool 1 cm above the water line. Rats were allowed to swim for 60 s. Time to reach the platform was recorded as escape latency. After completion of the last trial, rats were gently dried with a towel, kept warm for 1 h and returned to their home cages.

To assess memory consolidation, a probe trial was performed on day 8 and 9 after the 5 d acquisition tests. In this trial, the individual rat was placed into the pool as in the training trial, except that the hidden platform was removed from the pool. For these tests, time spent in the target quadrant within 60 s was recorded. The time spent in the target quadrant was taken to indicate the degree of memory consolidation that has taken place after learning. The time spent in the target quadrant was used as a measure of spatial memory. In probe trial, each rat was placed at a start position directly opposite to platform quadrant. Further, the number of times crossing over the platform site of each rat was also measured and calculated.

### Evaluation of blood glucose levels and body weight

Blood glucose levels were measured by Glucometer (Roche Diagnostics, Mannheim, Germany). In brief, blood samples were withdrawn from the rats using tail vein rupture method, and drop of blood was placed on the glucometer strip loaded in the glucometer for blood glucose determination. During the experiment, blood glucose levels and body weights were verified in the interim after the beginning of treatment.

### HPLC-UV method

The working system of HPLC-UV was used as following: mobile phase 0.01 mol/L NH_4_Ac: CH_3_OH (35:65, V/V), flow rate 1 mL/min, detection wavelength 247 nm. The pharmacokinetic parameters of ATZD2 (25 mg/kg, *i.p*) were obtained as followed: the AUC (0-∞) 14.58 ± 1.45 mg/L*h, Tmax 0.17 ± 0 h, *CLz*/F 1.73 ± 0.17 L/h/kg, *t*1/2 0.73 ± 0.075 h, Cmax 26.39 ± 1.02 μg/mL.

### Estimation of cytokines

After the MWM test, blood samples were taken from the abdominal aorta and left at room temperature for 1 h to allow complete clotting and were then centrifuged at 1,500 g for 15 min, followed by the separation of serum from the blood cells. Serum was aliquoted into centrifuge tubes and stored at –80°C until analysis. The levels of TNF-α, IL-1β and IL-10 in serum were determined using a specific enzyme immunoassay following the manufacturers’ instructions. Absorbance values were measured at 450 nm using an ELISA plate reader (Bio-Tek, USA). All samples were measured in triplicate.

### Measurement of GSH-Px, SOD, CAT, and MDA levels

After the evaluation of learning and memory, the rats were sacrificed under phenobarbital (40 mg/kg) anesthesia. The hippocampus was rapidly removed and weighed following transcardial perfusion with ice cold normal saline. The hippocampus was then homogenized in 4 volumes of 0.1 mol/L ice-cold phosphate buffer (pH 7.4) and centrifuged at 20,000 g for 30 min. The supernatants were used for determination of MDA, GSH-Px levels and the activities of SOD and CAT by spectrophotometry according to the manufacturer's instructions. Triplicate samples were analyzed for each time point.

### Western blot analysis

Hippocampus tissues were homogenized with ice-cold lysis buffer. The homogenate was centrifuged at 15,000 g for 5 min at 4°C. Protein was quantified by a BCA Kit and equal amounts of protein (30 μg) separated on 10% polyacrylamide gel followed by transferred onto an Immun-Blot PVDF membrane. The membrane was blocked for 1 h with 5% non-fat milk in Tris-Phosphate buffer containing 0.05% Tween 20 (TBS·T). It was further incubated overnight at 4°C with primary antibodies including anti-Aldose reductase (1:1,000), anti-HIF-1α (1:1,000), anti-Procaspase 3 (1:1,000) and anti-Bax (1:1,000), β-actin (1:10,000) served as a loading control. After three washes with TBS·T, membranes were further incubated with HRP-conjugated secondary antibodies for 1–2 h and followed by three TBS·T washes. The target protein signal was detected and digitalized using ECL and Image J program. The ratio of the integrated optical density of immunoblot bands of the detected protein to that of β-actin was used for statistical analysis. The density of target protein was expressed as the percent of control from five independent experiments.

### Statistical analysis

Results were expressed as mean ± SEM. The data were analyzed by two-way or one-way analysis of variance (ANOVA) followed by Bonferroni and Tukey's multiple comparison tests, respectively. Statistical significance was considered at *P* < 0.05 in all the cases.

## References

[1] Gispen WH, Biessels GJ (2000). Cognition and synaptic plasticity in diabetes mellitus. Trends Neurosci.

[2] Yaffe K, Falvey C, Hamilton N, Schwartz AV, Simonsick EM, Satterfield S, Cauley JA, Rosano C, Launer LJ, Strotmeyer ES, Harris TB (2012). Diabetes, glucose control, and 9-year cognitive decline among older adults without dementia. Arch Neurol.

[3] Biessels GJ, Deary IJ, Ryan CM (2008). Cognition and diabetes: a lifespan perspective. Lancet Neurol.

[4] Tuzcu M, Baydas G (2006). Effect of melatonin and vitamin E on diabetes-induced learning and memory impairment in rats. Eur J Pharmacol.

[5] Sima AA (2010). Encephalopathies: the emerging diabetic complications. Acta Diabetol.

[6] Rosen P, Nawroth PP, King G, Moller W, Tritschler HJ, Packer L (2001). The role of oxidative stress in the onset and progression of diabetes and its complications: a summary of a Congress Series sponsored by UNESCO-MCBN, the American Diabetes Association and the German Diabetes Society. Diabetes Metab Res Rev.

[7] Nishikawa T, Edelstein D, Du XL, Yamagishi S, Matsumura T, Kaneda Y, Yorek MA, Beebe D, Oates PJ, Hammes HP, Giardino I, Brownlee M (2000). Normalizing mitochondrial superoxide production blocks three pathways of hyperglycaemic damage. Nature.

[8] Burdo JR, Chen Q, Calcutt NA, Schubert D (2009). The pathological interaction between diabetes and presymptomatic Alzheimer's disease. Neurobiol Aging.

[9] Schram MT, Euser SM, de Craen AJ, Witteman JC, Frolich M, Hofman A, Jolles J, Breteler MM, Westendorp RG (2007). Systemic markers of inflammation and cognitive decline in old age. J Am Geriatr Soc.

[10] Ryan CM (2005). Diabetes, aging, and cognitive decline. Neurobiol Aging.

[11] Brownlee M (2001). Biochemistry and molecular cell biology of diabetic complications. Nature.

[12] Feinstein DL (2003). Therapeutic potential of peroxisome proliferator-activated receptor agonists for neurological disease. Diabetes Technol Ther.

[13] Nicolakakis N, Aboulkassim T, Ongali B, Lecrux C, Fernandes P, Rosa-Neto P, Tong XK, Hamel E (2008). Complete rescue of cerebrovascular function in aged Alzheimer's disease transgenic mice by antioxidants and pioglitazone, a peroxisome proliferator-activated receptor gamma agonist. J Neurosci.

[14] Abbatecola AM, Lattanzio F, Molinari AM, Cioffi M, Mansi L, Rambaldi P, DiCioccio L, Cacciapuoti F, Canonico R, Paolisso G (2010). Rosiglitazone and cognitive stability in older individuals with type 2 diabetes and mild cognitive impairment. Diabetes Care.

[15] Sato T, Hanyu H, Hirao K, Kanetaka H, Sakurai H, Iwamoto T (2011). Efficacy of PPAR-gamma agonist pioglitazone in mild Alzheimer disease. Neurobiol Aging.

[16] Nissen SE, Wolski K (2007). Effect of rosiglitazone on the risk of myocardial infarction and death from cardiovascular causes. N Engl J Med.

[17] Zhang LH, Li YB, Wan XM, Zhao Z (2011). Pharmacokinetics and tissues distribution of rosiglitazone in rats. [Article in Chinese] Huadong Shifan Daxue Xuebao.

[18] Han J, Zhao MG, Zhang J, Ma L, Fan G (2012). 5,12-Dimethyl-pyrazino-[1,2-a: 4,5-a’]dibenzimidazole-5,12-diium dichloride dihydrate. Acta Crystallogr Sect E Struct Rep Online.

[19] Han J, Zhang J, Yang Q, Zhao MG, Fan G (2011). 2-Chloro-methyl-1-methyl-1,3-benzimidazole. Acta Crystallogr Sect E Struct Rep Online.

[20] Ramasamy R, Goldberg IJ (2010). Aldose reductase and cardiovascular diseases, creating human-like diabetic complications in an experimental model. Circ Res.

[21] Vangeison G, Carr D, Federoff HJ, Rempe DA (2008). The good, the bad, and the cell type-specific roles of hypoxia inducible factor-1 alpha in neurons and astrocytes. J Neurosci.

[22] Fujisawa Y, Sasaki K, Akiyama K (1991). Increased insulin levels after OGTT load in peripheral blood and cerebrospinal fluid of patients with dementia of Alzheimer type. Biol Psychiatry.

[23] Akash MS, Rehman K, Chen S (2013). Role of inflammatory mechanisms in pathogenesis of type 2 diabetes mellitus. J Cell Biochem.

[24] Zhou J, Zhou S (2014). Inflammation: therapeutic targets for diabetic neuropathy. Mol Neurobiol.

[25] Ryan CM, Freed MI, Rood JA, Cobitz AR, Waterhouse BR, Strachan MW (2006). Improving metabolic control leads to better working memory in adults with type 2 diabetes. Diabetes Care.

[26] Stackman RW, Lora JC, Williams SB (2012). Directional responding of C57BL/6J mice in the Morris water maze is influenced by visual and vestibular cues and is dependent on the anterior thalamic nuclei. J Neurosci.

[27] Strachan MW, R D Lawrence Lecture 2010 (2011). The brain as a target organ in Type 2 diabetes: exploring the links with cognitive impairment and dementia. Diabet Med.

[28] Koekkoek PS, Kappelle LJ, van den Berg E, Rutten GE, Biessels GJ (2015). Cognitive function in patients with diabetes mellitus: guidance for daily care. Lancet Neurol.

[29] Moghaddam HK, Baluchnejadmojarad T, Roghani M, Khaksari M, Norouzi P, Ahooie M, Mahboobi F (2014). Berberine ameliorate oxidative stress and astrogliosis in the hippocampus of STZ-induced diabetic rats. Mol Neurobiol.

[30] Williamson JR, Chang K, Frangos M, Hasan KS, Ido Y, Kawamura T, Nyengaard JR, van den Enden M, Kilo C, Tilton RG (1993). Hyperglycemic pseudohypoxia and diabetic complications. Diabetes.

[31] Vedantham S, Thiagarajan D, Ananthakrishnan R, Wang L, Rosario R, Zou YS, Goldberg I, Yan SF, Schmidt AM, Ramasamy R (2014). Aldose reductase drives hyperacetylation of Egr-1 in hyperglycemia and consequent upregulation of proinflammatory and prothrombotic signals. Diabetes.

[32] Yagihashi S, Yamagishi SI, Wada Ri R, Baba M, Hohman TC, Yabe-Nishimura C, Kokai Y (2001). Neuropathy in diabetic mice overexpressing human aldose reductase and effects of aldose reductase inhibitor. Brain.

[33] Zhang M, Gao X, Bai SJ, Ye XM, Zhang J (2014). Effect of pioglitazone on expression of hypoxia-inducible factor 1alpha and vascular endothelial growth factor in ischemic hindlimb of diabetic rats. Eur Rev Med Pharmacol Sci.

[34] Li G, Lu WH, Ai R, Yang JH, Chen F, Tang ZZ (2014). The relationship between serum hypoxia-inducible factor 1alpha and coronary artery calcification in asymptomatic type 2 diabetic patients. Cardiovasc Diabetol.

[35] Di Domenico F, Perluigi M, Butterfield DA, Cornelius C, Calabrese V (2010). Oxidative damage in rat brain during aging: interplay between energy and metabolic key target proteins. Neurochem Res.

[36] Lee AY, Chung SS (1999). Contributions of polyol pathway to oxidative stress in diabetic cataract. FASEB J.

[37] Kyselova Z, Stefek M, Bauer V (2004). Pharmacological prevention of diabetic cataract. J Diabetes Complications.

[38] Fukui K, Omoi NO, Hayasaka T, Shinnkai T, Suzuki S, Abe K, Urano S (2002). Cognitive impairment of rats caused by oxidative stress and aging, and its prevention by vitamin E. Ann N Y Acad Sci.

[39] Chiu SL, Chen CM, Cline HT (2008). Insulin receptor signaling regulates synapse number, dendritic plasticity, and circuit function *in vivo*. Neuron.

[40] Apostolatos A, Song S, Acosta S, Peart M, Watson JE, Bickford P, Cooper DR, Patel NA (2012). Insulin promotes neuronal survival via the alternatively spliced protein kinase CdeltaII isoform. J Biol Chem.

[41] Butterfield DA, Di Domenico F, Barone E (2014). Elevated risk of type 2 diabetes for development of Alzheimer disease: a key role for oxidative stress in brain. Biochim Biophys Acta.

[42] Marioni RE, Strachan MW, Reynolds RM, Lowe GD, Mitchell RJ, Fowkes FG, Frier BM, Lee AJ, Butcher I, Rumley A, Murray GD, Deary IJ, Price JF (2010). Association between raised inflammatory markers and cognitive decline in elderly people with type 2 diabetes: the Edinburgh Type 2 Diabetes Study. Diabetes.

